# Clinical features and health-related quality of life in adult patients with mucopolysaccharidosis IVA: the Spanish experience

**DOI:** 10.1186/s13023-021-02074-y

**Published:** 2021-11-03

**Authors:** Pilar Quijada-Fraile, Elena Arranz Canales, Elena Martín-Hernández, María Juliana Ballesta-Martínez, Encarna Guillén-Navarro, Guillem Pintos-Morell, Marc Moltó-Abad, David Moreno-Martínez, Salvador García Morillo, Javier Blasco-Alonso, María Luz Couce, Ricardo Gil Sánchez, Elisenda Cortès-Saladelafont, Mónica A. López Rodríguez, María Teresa García-Silva, Montserrat Morales Conejo

**Affiliations:** 1 Unidad de Enfermedades Mitocondriales y Enfermedades Metabólicas Hereditarias, Servicio de Pediatría, Hospital Universitario 12 de Octubre, CSUR Enfermedades Metabólicas, MetabERN, Instituto de Investigación Sanitaria Hospital 12 de octubre (imas12), CIBERER, Madrid, Spain; 2 Servicio de Medicina Interna, CSUR Enfermedades Metabólicas, MetabERN, Instituto de Investigación Sanitaria Hospital 12 de octubre (imas12), Hospital Universitario 12 de Octubre, Madrid, Spain; 3 Sección de Genética Médica, Hospital Clínico Universitario Virgen de la Arrixaca, IMIB-Arrixaca, Universidad de Murcia, Murcia, Spain; 4 CIBERER-ISCIII, Madrid, Spain; 5 Division of Rare Diseases, Reference Center for Hereditary Metabolic Disorders (CSUR, MetabERN, MetabXUEC), University Hospital Vall d’Hebron, Barcelona, Spain; 6 Lysosomal Storage Disorders Unit, The Royal Free Hospital NHS Foundation Trust and University College London, London, UK; 7 Unidad de Enfermedades Autoinmunes y Minoritarias, Servicio de Medicina Interna, Hospital Virgen del Rocío, Sevilla, Spain; 8 Unidad de Gastroenterología y Nutrición Infantil, Grupo IBIMA Multidisciplinar Pediátrico, Hospital Regional Universitario de Málaga, Málaga, Spain; 9 Unidad de Diagnóstico y Tratamiento de Enfermedades Metabólicas Hereditarias, Hospital Clínico Universitario de Santiago, IDIS, MetabERN, CIBERER, Santiago de Compostela, Spain; 10 Hospital Universitario La Fe, Valencia, Spain; 11 Inborn Errors of Metabolism and Paediatric Neurology Unit, Paediatric Department, Hospital Universitari Germans Trias i Pujol, Badalona, Spain; 12 Servicio de Medicina Interna, CSUR Enfermedades Metabólicas Hereditarias, Hospital Universitario Ramón y Cajal, Madrid, Spain

**Keywords:** Elosulfase alfa, Health-related quality of life, Mobility, Morquio A syndrome, Mucopolysaccharidosis IVA

## Abstract

**Background:**

Mucopolysaccharidosis (MPS) IVA or Morquio A syndrome is a progressive and disabling disease characterized by a deficiency of the enzyme *N*-acetylgalactosamine-6-sulphate sulphatase. Its clinical presentation is very heterogeneous and poorly understood in adults.

The aim of this study was to describe the clinical manifestations of MPS IVA in adult patients in Spain and to assess their health-related quality of life (HRQoL).

**Results:**

Thirty-three patients from nine reference centres participated in the study. The median age was 32 (interquartile range [IQR]: 20.5–40.5) years. The phenotype was classical in 54.5% of patients, intermediate in 33.3% of patients, and non-classical in 12.1% of patients. The most common clinical manifestation was bone dysplasia, with a median height of 118 (IQR: 106–136) cm. Other frequent clinical manifestations were hearing loss (75.7%), ligamentous laxity (72.7%), odontoid dysplasia (69.7%), limb deformities that required orthopaedic aids (mainly hip dysplasia and genu valgus) (63.6%), and corneal clouding (60.6%). In addition, 36.0% of patients had obstructive sleep apnoea/hypopnoea syndrome and 33.3% needed non-invasive ventilation. Cervical surgery and varisation osteotomy were the most common surgical interventions (36.4% each). Almost 80% of patients had mobility problems and 36.4% used a wheelchair at all times. Furthermore, 87.9% needed help with self-care, 33.3% were fully dependent, and 78.8% had some degree of pain. HRQoL according to the health assessment questionnaire was 1.43 (IQR: 1.03–2.00) in patients with the non-classical phenotype, but 2.5 (IQR: 1.68–3.00) in those with the classical phenotype. Seven patients were initiated on enzyme replacement therapy (ERT), but two of them were lost to follow-up. Lung function improved in four patients and slightly worsened in one patient. The distance achieved in the six-minute walk test increased in the four patients who could perform it. HRQoL was better in patients treated with elosulfase alfa, with a median (IQR) of 1.75 (1.25–2.34) versus 2.25 (1.62–3.00) in patients not treated with ERT.

**Conclusions:**

The study provides real-world data on patients with MPS IVA. Limited mobility, difficulties with self-care, dependence, and pain were common, together with poor HRQoL. The severity and heterogeneity of clinical manifestations require the combined efforts of multidisciplinary teams.

## Background

Mucopolysaccharidosis (MPS) IVA or Morquio A syndrome (OMIM 253000) is a progressive and disabling disease. It is an inherited lysosomal disorder caused by pathogenic variants in the *GALNS* gene, which codifies the *N*-acetylgalactosamine-6-sulphate sulphatase enzyme. According to data from the Human Gene Mutation Database Professional, 348 pathogenic variants had been reported until August 2019 [1], and more than 70% were missense/nonsense mutations [2]. This disorder leads to impaired lysosomal degradation of the glycosaminoglycans (GAGs) keratan sulphate and chondroitin-6-sulphate, which alter cell function. The clinical manifestations of MPS IVA reflect the tissue distribution of keratan sulphate and chondroitin-6-sulphate [3].

Although bone dysplasia is the most typical feature, it is a multisystemic disease, with both skeletal and non-skeletal symptoms. The clinical presentation of MPS IVA is widely heterogeneous, from the classical phenotype, through the intermediate phenotype, to the non-classical phenotype [4]. This phenotypic variability is related to the high diversity of *GALNS* mutations [5, 6]. The traditional classification of MPS IVA is subjective, and the disease should be considered as a continuum [7]. Skeletal symptoms include bone deformities, short stature, and gait disturbances, as well as genu valgum, ligamentous laxity and kyphosis, among others [3]. Non-skeletal symptoms are due to GAG storage in areas such as the ear, cornea, teeth, cardiac valves, upper airways, and liver [4].

The diagnosis of MPS IVA is initiated by the recognition of clinical and radiological features, and then the following laboratory tests can be performed: a biochemical test (total urine GAGs), an enzymatic test (N-acetylgalactosamine-6-sulphate sulphatase activity) and molecular analysis [2, 3].

Treatment of MPS IVA was symptomatic only until the availability of ERT with elosulfase alfa (Vimizim®, BioMarin Pharmaceutical Inc., San Rafael, CA, USA), which was approved by both the US Food and Drug Administration and the European Medicines Agency in 2014. In a long-term study, endurance improved in patients treated with elosulfase alfa [8]: 26 patients (17 adults and nine patients younger than 18 years) showed general improvement in both endurance in the six-minute walk test (6MWT) and the performance of activities of daily living (ADL) during the first year of treatment. These improvements increased or stabilized over a follow-up of up to seven years [9].

In Spain, paediatric patients with MPS IVA are managed by paediatric specialists in clinics devoted to congenital metabolic diseases and/or skeletal dysplasias. However, it has proved difficult for paediatric patients with MPS to access appropriate long-term follow-up on reaching adulthood, despite several initiatives to improve transition from paediatric to adult care for these patients [10]. Knowledge on the clinical evolution of MPS IVA in adults is limited, partly because of the low prevalence of the disorder, and partly because of the absence of reference centres in Spain in the past. The recent creation of such reference centres for adult patients with inherited metabolic diseases in Spain will improve our understanding of the natural history of MPS IVA.

It has been suggested that hematopoietic stem cell transplantation (HSCT) could be useful in MPS IVA. However, based on current evidence, it cannot be recommended [11].

The quality of life of patients with rare diseases has not been extensively assessed [12], but one large study showed that health-related quality of life (HRQoL) was lower in patients with rare diseases than in patients with other chronic diseases [13].

The objectives of the present study were therefore to describe the clinical characteristics of adult patients with MPS IVA in Spain, and to assess the burden of disease and the HRQoL in these patients.

## Results

### Sociodemographic and diagnostic data

Conducted between May 2018 and May 2019, the study included 33 adult patients from nine tertiary hospitals in Spain. All the questionnaires sent were completed and returned.

Table 1 shows the sociodemographic and diagnostic data of the study population. Patients aged between 16 and 65 years (median 32), and more than half (51.5%) were male.

**Table 1:** Sociodemographic and diagnostic variables in a population of adult patients with MPS IVA in Spain

Variable	Value (N = 33)
*Age, median (IQR), years*	32 (20.5–40.5)
*Sex, n* (*%*)	
Men	17 (51.5)
Women	16 (48.5)
*Height, median (range) (cm)*	118 (106–136)
Men	126 (113.7–142.5)
Women	116 (105.2–124.7)
*Weight, median (range) (kg)*	34 (28–40)
Men	35.5 (30.9–49.7)
Women	31 (25.4–36.7)
*Phenotype*	
Classical	18 (54.5)
Intermediate	11 (33.3)
Non-classical	4 (12.1)
*BMI* (*kg/m*^*2*^)	
Classical phenotype	21.36 (18.23–25.30)
Intermediate phenotype	24.68 (23.36–31)
Non-classical phenotype	20.03 ( 16.31–26)
*Consanguinity history, n* (*%*)	3 (9)
*Patients with affected siblings, n* (*%*)	12 (36.6)
*Age at diagnosis, years* (*IQR*)	5.5 (2–12.6)
*GAGs, mg/mmol creatinine* (*IQR*)	22.60 (6.81–29.01)
*Common GALNS mutations, n* (*%*)	
c.1156C > T	8 (24.2) [6 patients were homozygous]
c.901G > T	4 (12)
c.761A > G	2 (6) [both patients were homozygous]
*First symptom at diagnosis, n* (*%*)	
Bone dysplasia	8 (24.2)
Kyphoscoliosis	5 (15.1)

GAGs, glycosaminoglycans (keratan sulphate and chondroitin-6-sulphate); IQR, interquartile range; MPS, mucopolysaccharidosis

Urinary GAGs were measured in 18 patients (54.5%), either at diagnosis (16 patients, 48.5%) or later (2 patients, 6.1%), and were found to be elevated in all them. In addition, enzyme activity was analysed in 16 patients (48.5%). Although measurement methods and reference values differed between laboratories, enzyme activity was reduced in all these patients (data not shown).

Genetic variants of GALNS were analysed in 16 patients (48.5%). The most common GALNS mutation was the missense mutation c.1156C > T, which was found in eight (24.2%) patients (six of whom were homozygous), all with classical phenotype. Another frequent GALNS mutation was c.901G > T, found in 4 patients (12.1%), with 2 patients with classical phenotype and 2 patients with intermediate phenotype. It was also common the mutation c.761A > G, found in two patients (6.1%) (both homozygous and with intermediate phenotype).

Weight and length at birth were normal in the 11 patients with data available. Patients had been diagnosed during childhood and the most common clinical feature at diagnosis was bone dysplasia. More than half (54.5%) the patients had the classical phenotype (Table 1). These patients were shorter than those with intermediate or non-classical phenotypes were.

### Clinical manifestations

**Fig. 1:**
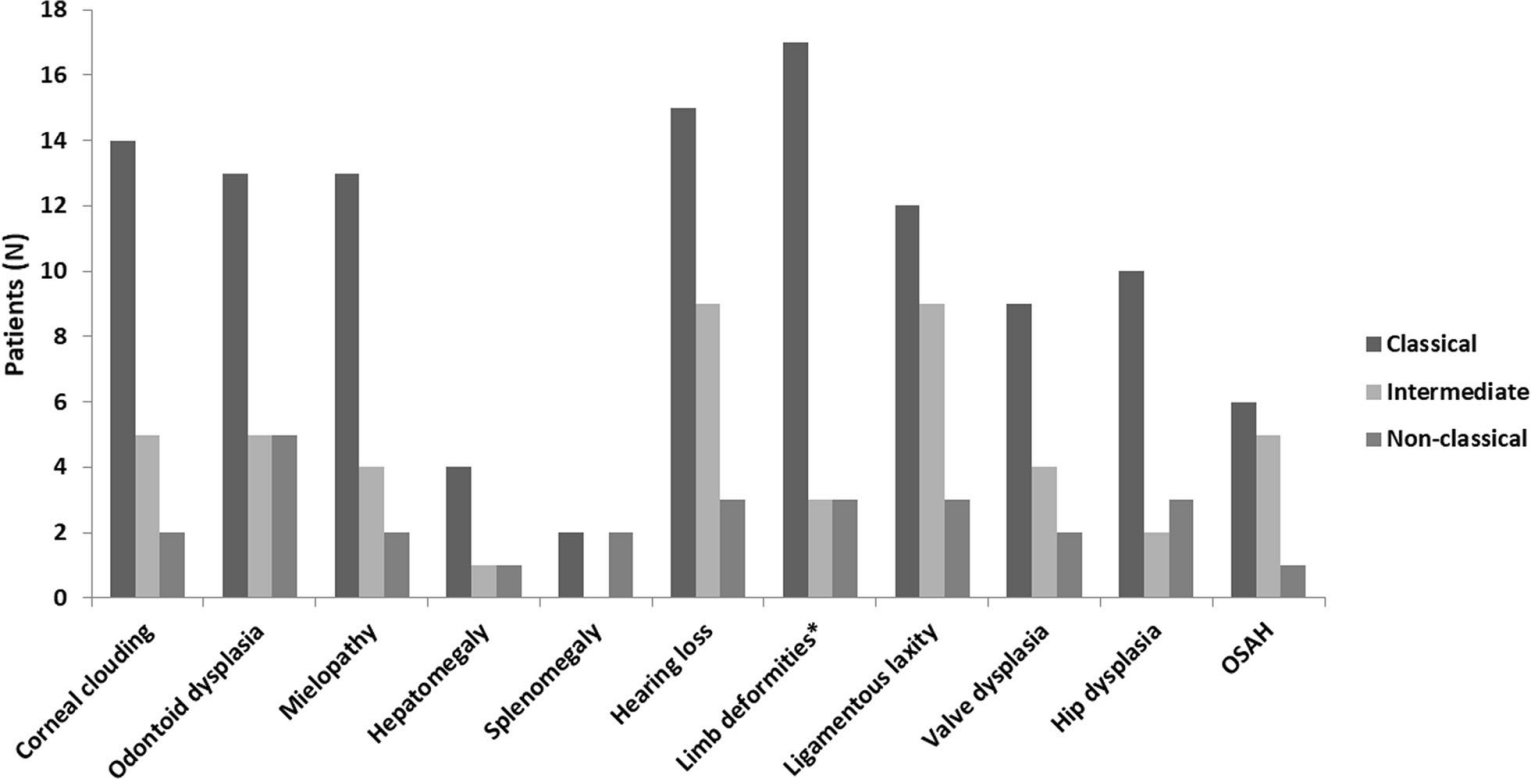
Common clinical manifestations other than bone dysplasia by phenotype in adult patients with MPS IVA in Spain. *Deformities that required orthopaedic aids (mainly hip dysplasia and genu valgus). MPS, mucopolysaccharidosis; OSAH, obstructive sleep apnoea/hypopnoea syndrome

All patients had typical clinical manifestations of Morquio A disease: skeletal dysplasia, including dwarfism with short trunk and neck; pectus carinatum; genu valgum; and joint deformities. Height and weight were low (Table 1). Figure 1 shows common clinical manifestations other than bone dysplasia according to the phenotypes.

### Lung function

Lung function was assessed in only 22 patients, as some were unable to perform the tests because of difficulty with the technique. Basal oxygen saturation was measured in 20 patients and found to be normal in all of them. However, obstructive sleep apnoea-hypopnoea syndrome was shown in 12 patients (36.4%), 11 of whom needed non-invasive ventilation (biphasic positive airway pressure in one patient; nocturnal biphasic positive airway pressure in four patients; and nocturnal continuous positive airway pressure in six patients). Another patient required tracheostomy.

With regard to lung function tests, the median forced expiratory volume in 1 s was 840 (435–1470) mL and the median forced vital capacity was 1020 (515–1675) mL.

FEV1/FVC expressed as percentage was 77.50 (72.70–84.69) in patients with classical phenotype, 89.98 (86.71–92) in patients with intermediate phenotype, and 89.98 (86.1–92) in patients with non-classical phenotype.

### 6MWT

Most patients were unable to perform the 6MWT, with only eight patients completing the test. The median walking distance was 263.5 (140.3–436.5) m.

### Results in patients treated with ERT

Seven patients, all them with classical phenotype, were initiated on ERT therapy. However, 2 patients were lost to follow-up. In 5 patients, treatment was started younger than 20 years of age. The dose administered was 2 mg/kg by intravenous infusion over 3.5–4.5 h or longer.

At the last visit of the study, lung function had improved in 4 patients and slightly worsened in 1 patient. The walking distance in the 6MWT increased in the four patients who could perform the test (Table 2). All patients treated with ERT reported a reduction in pain, although height was similar in these patients to that of patients who did not receive ERT.

**Table 2:** Results in patients with MPS IVA treated with elosulfase alfa

Patient number	Time on ERT, years	FVC (mL)		FEV_1_ (mL)		6MWT (m)	
		Before ERT	Last visit	Before ERT	Last visit	Before ERT	Last visit
1	1	1663	1610	1361	1358	125	150
2	1	1430	1452	1148	1154	39	86.4
3	6	938	1318	896	1068	32	424
4	4	2460	2690	2180	2360	–	–
5	4	780	870	680	730	302	351

6MWT, six-minute walk test; ERT, enzyme replacement therapy; FEV_1_, forced expiratory volume in 1 s; FVC, forced vital capacity

### Surgical interventions

**Fig. 2:**
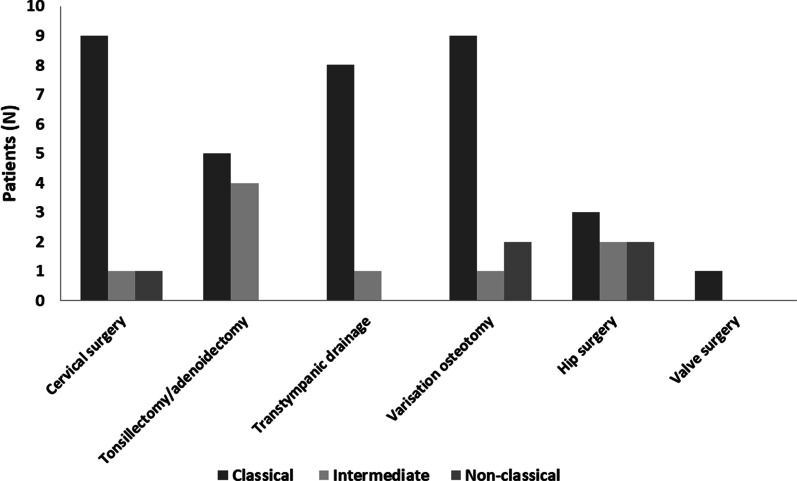
Surgical interventions by phenotypes in adult patients with MPS IVA in Spain. MPS, mucopolysaccharidosis

The two most common surgical interventions were cervical surgery and knee varisation osteotomy. Other surgical interventions were surgery for hip dysplasia, adenoidectomy and/or tonsillectomy, transtympanic drainage, and cardiac valve surgery (Fig. 2). Results of surgical interventions were not satisfactory in all patients.

### Burden of disease

Table 3 gives details of the burden of disease. According to the results of the study questionnaire, almost 80% of patients had mobility problems, with more than 40% being severe problems. More than 65% of patients used a wheelchair, with more than one-third of patients needing to use one at all times. Almost all patients needed help with self-care, and one-third of patients were unable to take care of themselves. More than 75% of patients needed help to perform ADL, with one-third being fully dependent.

**Table 3:** Burden of disease in a population of adult patients with MPS IVA in Spain

Variable	Value, n (%) (N = 33)
*Mobility problems*	26 (78)
Mild	9 (27.3)
Moderate	3 (9)
Severe	14 (42.2)
*Wheelchair use*	22 (66.7)
Always	12 (36.4)
*Need help with self-care*	29 (87.9)
Fully dependent	11 (33.3)
*Need help with daily activities*	25 (75.7)
Mild help	6 (18.2)
Moderate help	8 (24.2)
Fully dependent	11 (33.3)
*Pain*	26 (78.8)
Mild	13 (39.4)
Moderate	9 (27.3)
Severe	4 (12.1)
*Anxiety/depression*	8 (24.2)

MPS, mucopolysaccharidosis

Also according to the results of the study questionnaire, more than 80% of patients reported some degree of pain and almost 25% of patients had anxiety/depression. Pain was worse in patients who were permanently in a wheelchair. Only five patients had a job (four of them working part-time) and only six patients lived independently.

Twenty-one patients completed the health assessment questionnaire (HAQ). The median score was 2.12 (range 1.62–3.00). However, HRQoL scores differed according to phenotype: the median HAQ score was 1.43 (range 1.03–2.03) in patients with non-classical phenotype, but 2.44 (range 1.62–3.00) and 2.5 (range 1.68–3.00) in patients with the intermediate and classical phenotypes, respectively. Furthermore, HRQoL was generally better in patients treated with ERT, with a median of 1.75 (range 1.25–2.34) versus 2.25 (range 1.62–3.00) in patients who did not receive ERT.

## Discussion

We gathered data from 33 adult patients with MPS IVA. As the questionnaire return rate was 100%, this number of patients is probably very close to the whole population of MPS IVA adult patients in Spain, although there may be a number of patients who are undiagnosed.

The median age at diagnosis was 5 years, which concurs with data in the International Morquio A Registry in 2007 [4]. More than one-third of our patients had affected siblings, possibly because genetic counselling and prenatal testing were more difficult to obtain years ago than nowadays, or even unavailable.

More than 50% of our patients had the classical phenotype. We did not examine the relationship between phenotype and *GALNS* mutations, and genetic analysis was available for only 16 patients. However, we found two *GALNS* mutations that have been associated with classical phenotype (c.1156C > T and c.901G > T) [14]. It would be interesting to assess such relationship in a future study with more complete genetic data available.

### Clinical manifestations

With regard to the clinical manifestations of MPS IVA, height was under the third percentile in most patients—short stature is common in MPS IVA patients [4]. As expected, length and weight were normal at birth.

Another common clinical manifestation was ligamentous laxity, which can complicate personal care and activities such as getting dressed. It can also make writing difficult, which can be a problem for work or study.

Other non-skeletal manifestations, such as corneal clouding and respiratory compromise, were frequent in our patient population, correlating with findings in other studies [4, 15]. We found that up to one-third of patients needed non-invasive ventilation. This finding highlights the relevance of periodic respiratory check-ups in patients with MPS IVA.

Hearing loss is also common in MPS IVA. Patients suffer frequent otitis and middle and inner ear abnormalities, which cause mixed hypoacusis at around 5 years of age [3]. More than 75% of patients with MPS IVA in our study had hearing loss, a percentage similar to that found in the Morquio A Clinical Assessment Program study [15].

### ERT

In our study, five of the seven patients who began ERT did so before the age of 18 years under an early access programme [16]. Two patients left the country and were lost to follow-up, meaning we had data from only five patients treated with elosulfase alfa. This small number of patients meant that it was not possible to perform an in-depth analysis of the potential effects of ERT. However, in one study of 37 adult patients with MPS IVA treated for 120 weeks with elosulfase alfa, endurance increased and performance of ADL improved, without changes in pulmonary function [17]. However, in 10 patients with more severe disease and limited mobility treated with elosulfase alfa for 48 weeks, results were inconsistent because of clinical heterogeneity of patients, severe deformities, and the small sample size [18]. Nevertheless, in a study with 5-year follow-up, elosulfase alfa prevented disease progression [19]. Moreover, performance of ADL improved after 120 weeks of ERT in 170 patients [20].

### Surgery

The percentages of surgical intervention were similar to those found in the International Morquio A Registry in 2007, in which 50% of patients underwent cervical surgery, 30% ear surgery, 26% limb surgery and 25% hip surgery [4]. Orthopaedic surgery in patients with MPS IVA should be considered individually, assessing risk, timing, pain, and patient preferences [11].

### Burden of disease

Patients with MPS IVA had reduced mobility [4, 15, 21]. In our study, almost 80% of patients had problems for walking, and more than 65% needed a wheelchair some or all the time. It should be noted that wheelchair use has been related to worse HRQoL [22]. Furthermore, as shown in our study, patients with MPS IVA need help with self-care and performing ADL [22].

Another common symptom in patients with MPS IVA is pain, which is related to musculoskeletal problems. Pain can affect the spine, the upper and lower extremities, and the head and neck, and can interfere with mobility and ADL. In our study, almost 80% of patients reported some degree of pain. Similarly, in a patient-reported outcomes survey, 74% of adults reported joint pain [22]. Pain was more severe in wheelchair users than in non-users and was worse in patients who used a wheelchair all the time compared with those who used a wheelchair intermittently, in contrast with other published results [22].

Psychological symptoms can go unnoticed because of the severity of physical symptoms. However, many patients with MPS have some degree of psychological disturbance [23], with more than 50% of patients with MPS IVA having psychological symptoms in one study [24]. We found that 20% of patients symptoms of anxiety, but we did not use a specific questionnaire for mental health. Therefore, this percentage is not conclusive.

The high burden of disease in our study could be partly because 60% of our patients had not been cared for at paediatric reference units when they were children/teenagers, being managed instead at trauma and orthopaedic clinics because of the bone dysplasia, but without specific follow-up of the other disease features. In addition, the transition from paediatric to adult units is not always well managed in Spain.

Various factors relating to surgery are also probably implicated in the high disease burden. First, the number of surgical procedures was insufficient. Second, surgical outcomes were not as good as expected or desired, perhaps because some procedures were performed at non-specialist centres. In general terms, these patients may not have received optimal healthcare because of the severity of their disease and the absence of referral to specialist centres. This, together with a lack of co-operation from some patients not happy with the multiple examinations, may also be the reason for the large amount of data missing.

Finally, earlier onset of ERT probably leads to a better disease course. However, the beneficial effect of elosulfase alfa has still to be confirmed in long-term studies inpatients treated from early age [11].

### HRQoL

Disease progression impairs HRQoL in patients with MPS IVA. The median score of HAQ in the present study was 2.12; as HAQ scores can range between 0 (no disability) and 3 (maximum disability) [22], this result shows that patients with MPS IVA had an important disability. Moreover, as we confirmed in our study, HRQoL is worse in patients with the intermediate or classical phenotypes. Many factors contribute to a poor HRQoL: mobility, endurance, problems performing ADL, dependence on caregivers, frequent surgical interventions, fatigue and pain. Maintaining functionality and mobility, as well as pain control, will probably improve HRQoL in patients with MPS IVA [21].

Unemployment is related to a poorer HRQoL. Only 15% of our patients had a job, and only one patient worked full-time. In an international study in patients with MPS IVA, HRQoL was statistically significantly better in employed patients [22]. This difference could be due not only to higher psychological wellness, but also to a better physical state.

In this study, HRQoL, as measured by the HAQ score, was higher in patients treated with elosulfase alfa than in those not treated with ERT. Because of the small number of patients, we did not perform any statistical analysis, but we could hypothesise that ERT improved mobility, and thence HRQoL. A further comparison of 6MWT results in a larger number of ERT treated and untreated patients would be necessary.

It is recommended that the disease burden be assessed each year. At the annual visit, the physician in charge should evaluate HRQoL, fatigue, ADL, pain severity, and use of wheelchair or walking aids [22]. In addition, psychological health deserves more attention and should be assessed regularly [24].

### Study limitations

It was a multicentre retrospective study and some data were missing. Especially in older patients, some data were not recorded or were lost. Regarding diagnosis test, urinary GAGs, enzyme activity and GALNS mutations were not assessed in all patients. The reasons were that these test were not available or easily accessed when middle age or old patients were diagnosed. In addition, urinary GAGs were measured in different laboratories with different references values. Furthermore, available tests were not the same for all laboratories and hospitals.

Classification of patients as classical, intermediate and non-classical phenotypes is not current practice. However, as it is stated above, the disease should be considered as a continuum [7]. Moreover, this classification allowed a better comparison between patients. In addition, although height is not the only abnormality that could be assessed, it is an objective measure that reflects the severity of bone dysplasia. Other authors have also categorized patients in these three groups by height [3, 7, 9].

We did not analyse GAG levels because measurements were performed at different laboratories with different cut-off points and different measurement units. However, all laboratories were specialized centres that were validated for the study of the MPS IVA.

As for ERT, elosulfase alfa has been approved by the Spanish Agency of Medicines and Medical Devices for the treatment of MPS IVA, but it is not yet on the market in Spain. Therefore, access to ERT is limited and only seven patients in the study received elosulfase alfa, all them on compassionate use.

## Conclusions

Despite the limitations of a retrospective study, we were able to assess many variables. We therefore believe that our results are representative of the clinical status of adult patients with MPS IVA in Spain. Our study contributes to a better knowledge of the real-world clinical evolution of patients with MPS IVA into adulthood. The impaired mobility, hearing loss, pain, and poor HRQoL experienced by patients with MPS IVA demand the combined efforts of multidisciplinary teams. Future studies of the correlation between genotype and phenotype, as well as studies of biomarkers to evaluate disease progression and response to treatment, will help to provide better healthcare for patients with MPS IVA.

## Methods

We conducted an observational, retrospective, descriptive study of adult patients with MPS IVA in Spain. Patients were included if they were aged 16 years or older, had been diagnosed with MPS IVA, and were being followed at Spanish reference centres for inherited metabolic diseases. Diagnosis of MPS IVA was based in clinical criteria and GAGs, enzyme activity and/or genetic study. Exclusion criteria were being unable to access medical records; not being willing to participate in the study.

We developed a questionnaire that included demographic, diagnostic and clinical variables. Sex, age, consanguinity, and family history were the main demographic variables, while the diagnostic variables included age at diagnosis, weight and length at birth, GAG levels in urine, and enzyme activity and/or molecular analysis. The clinical variables were phenotype, skeletal abnormalities, myelopathy (yes/no), history of neurosurgery, corneal clouding (yes/no), odontoid dysplasia (yes/no); ear, nose and throat abnormalities; enlarged liver (yes/no); enlarged spleen (yes/no); respiratory function; and cardiovascular abnormalities and history of cardiovascular surgery. Pain was assessed with a visual analogue scale (VAS) from 0 (no pain) to 10 (extreme pain). The VAS classified pain as mild (1–3), moderate (4–6) and severe (7–10). We also recorded the results of the 6MWT and mobility, ADL, self-care, pain, and anxiety tests. In addition, we assessed HRQoL with the Spanish version of the HAQ, the scores for which range between 0 (no disability) and 3 (maximum disability) [25, 26]. This version is the adaptation for the Spanish population of the HAQ disabilty and pain scales [27] and was obtained from the Spanish Society of Rheumatology web page. The questionnaire was emailed to specialist physicians caring for adult patients with MPS IVA at all Spanish reference centres for inherited metabolic diseases.

As all patients were adults, they were classified according to their phenotype: classical (final height below 120 cm, with -9.43 SD compared with healthy adult height), intermediate (final height higher than 120 cm but lower than 140 cm, with − 6.11 SD), and non-classical (final height higher than 140 cm) [9]. The study was approved by the Research Ethics Committee of the University Hospital 12 de Octubre, Madrid, Spain (CEI number 17/447).

### Statistics

We performed a descriptive analysis using SPSS software v. 21(IBM, New York, NY, USA).

## Data Availability

The datasets used and/or analysed during the current study are available from the corresponding author on reasonable request.

## Abbreviations

6MWT: Six-minute walk test
ADL: Activities of daily living
ERT: Enzyme replacement therapy
GAG: Glycosaminoglycan
HAQ: Health assessment questionnaire
HRQoL: Health-related quality of life
MPS: Mucopolysaccharidosis
